# Psychometric properties of the Chinese version of the self-care scale for older adults undergoing hip fracture surgery: A translation and validation study

**DOI:** 10.3389/fpubh.2023.1119630

**Published:** 2023-03-15

**Authors:** Chen Zheng, FangLin Liu, Yan Zheng, Ping Chen, MingYue Zhou, Huijun Zhang

**Affiliations:** ^1^School of Nursing, Jinzhou Medical University, Jinzhou, Liaoning, China; ^2^Operating Room of People's Hospital, Xinzhou, Shanxi, China

**Keywords:** hip fracture, old age, self-care, factor analysis, psychological measurement assessment

## Abstract

**Objective:**

The purpose of this study was to translate and verify the reliability and validity of the Chinese version of the self-care scale for older adults undergoing hip fracture surgery.

**Methods:**

A total of 502 older adult/adults patients after hip fracture surgery were recruited from Liaoning, Shanxi, and Beijing, China. The reliability of the Chinese version of the scale was measured by internal consistency, split-half reliability, and retest reliability, and the validity was evaluated by the content validity index and structure validity index.

**Results:**

The Chinese version of the HFS-SC scale had a Cronbach's alpha coefficient of 0.848, and the Cronbach's alpha coefficients for the five dimensions ranged from 0.719 to 0.780. The split-half reliability of the scale was 0.739, and the retest reliability was 0.759. The content validity index (S-CVI) was 0.932. The five-factor structure, supported by the eigenvalues, total variance explained, and the scree plot accounted for 66.666% of the total variance. In confirmatory factor analysis, the model fit results were as follows, X2/df = 1.847, GFI = 0.914, AGFI = 0.878, PGFI = 0.640, IFI = 0.932, TLI = 0.912, CFI = 0.931, RMSEA = 0.058, PNFI = 0.679. The indicators of the model's fit were within reasonable bounds.

**Conclusion:**

The Chinese version of the self-care scale for older adults undergoing hip fracture surgery has suitable reliability and validity. The scale can be used to assess the level of older adult/adults self-care in China following hip replacement surgery and serves as a useful benchmark for identifying potential intervention targets to raise the level of older adult/adults self-care following hip replacement surgery.

## 1. Introduction

Population aging, which is a universal phenomenon with characteristics of huge scale, depth, and speed, is the inevitable outcome of demographic transformation (1, 2). In addition to a sizable older population, age-related diseases are becoming more common and prevalent, which is having a negative impact on worldwide public health (3). With the development of an aging society, the increasing number of fractures has become one of the public health challenges affecting the healthy life span and quality of life of the older adult/adults. Hip fracture is one of the most serious types of fractures (4). Older people are the main patient group for common clinical hip fractures. The main risk factors for hip fracture are osteoporosis and falls. The older adult/adults are more prone to hip fractures from minor violence due to increased bone fragility and deterioration in all aspects of physical function (5). Studies have shown that the overall incidence of hip fractures is on the rise in various countries. With a one-year mortality rate of up to 30%, hip fracture patients are one of the more common sources of fracture morbidity and mortality in the older adult/adults. Hip fractures not only result in direct mortality but also account for about 25% of all-cause mortality (6, 7). It has the characteristics of high incidence, high disability rate, and high mortality, which seriously affects the health and quality of life of the older adult/adults. The hip fractures will not only reduce life expectancy, but also reduce the activity level and daily living ability of older adult/adults patients, which brings a heavy economic and mental burden to society and families, causing serious public health problems (8).

Geriatric hip fracture is a common and severe type of fracture that occurs in people 65 and older. Specifically, it refers to the continuous fracture at the junction of the femur and pelvis, which is anatomically divided into intracapsular and extracapsular fractures, including femoral neck fractures, trochanteric region, and fractures within 5 cm below the lesser trochanter (9). Numerous studies have shown that age is an important predictor of the development and progression of hip fractures. In 2050, 6.26 million hip fractures are expected to occur worldwide, with people 65 and older accounting for 80% of cases (10, 11). In 2015, the incidence of hip fractures among people over 74 years old in China reached 5.42%. Hip fractures are becoming more common, and the high cost of treatment has put a significant financial strain on families and society as a whole (12–14).

According to studies, surgically treating hip fractures provides clear survival advantages that can help increase survival rates and lower death (5, 15). As a result, surgical therapy is preferred by the majority of hip fracture patients. older adult/adults people with hip fractures typically have complex medical problems and more chronic illnesses. Following surgery, they are traumatized and frail, frequently with a reduction in walking ability, which raises their risk of falling and further injury (16). These elements will make nursing work more challenging (17). older adult/adults HF patients' caring responsibilities are primarily handled by spouses, adult children, etc. once they are released from the hospital. The initial phases of rehabilitation and exercise place a significant burden on these unofficial caregivers. The quality of life of patients is severely impacted by the lengthy HF treatment and recovery cycle, as well as the absence of professional rehabilitation assistance after discharge, which has negative consequences on rehabilitation outcomes and other circumstances (4). For the rehabilitation of older adult/adults individuals after hip fracture surgery, post-operative scientific and appropriate self-care is crucial.

Self-care is the action people do to support clinical therapy, and maintain or advance their health, to continually developing patients' capacity for self-care and self-care to reach health levels and return to normal (18). Orem proposed the self-care idea at the beginning of the 1970s, urging patients to use all resources to maintain their current level of health and hasten the healing of illnesses (19). After a hip fracture, older adult/adults people's ability to take care of themselves is crucial to their recovery. According to studies, Orem self-care mode can significantly increase patients' illness cognition, self-care skills, and hip function when it comes to nursing older adult/adults patients with hip fractures. Patients can improve their health following surgery, significantly lessen the traumatic stress reaction, and ultimately lower the overall incidence of problems by modifying their negative behaviors (20). The development of self-care abilities is important for disease treatment and symptom control, as well as for the postoperative health of patients. Therefore, self-care is a useful strategy for managing health and encouraging active recovery in older patients who have undergone hip fracture surgery, which merits discussion.

The capacity of older patients to care for themselves following hip fracture surgery is drawing more and more attention as the state of medicine evolves. Therefore, research into older Chinese people with hip fractures' capacity for self-care is important. The development of a tool to assess the level of self-care after hip fracture in the older adult/adults Chinese population is seen as essential to better understanding life after hip fracture in the older adult/adults Chinese population due to the high prevalence and significant negative effects of hip fractures in the geriatric population. The self-care scale for older adults undergoing hip fracture surgery (HFS-SC) developed by Jeon et al. was used to measure the ability of older people to self-care after hip fracture surgery (21). The scale was logically constructed for the old population, taking into full account the degree of disease prevention and cognition of older adult/adults hip fracture patients after surgery, based on the background of high morbidity and extended duration of disease in the older adult/adults hip fracture population. In the Korean population, the scale has been shown to have good psychometric qualities.

The study aims to translate and cross-culturally debug the scale, introduce the Korean version of the HFS-SC into China, and evaluate the reliability and validity of the translated scale in older adult/adults Chinese patients after hip fracture surgery.

## 2. Methods

### 2.1. Design and participants

This study was undertaken from August 2022 to December 2022 to evaluate the degree of self-care of older adult/adults hip fracture patients after surgery and to measure their psychometric characteristics through a cross-sectional study. The sample size was determined in accordance with the general rules of the faction-analysis program, which require the recruitment of at least ten respondents per project, but a larger sample is desirable (22). To ensure the accuracy of exploratory factor analysis and confirmatory factor analysis, 15 respondents for each project should be recruited to participate in the study. In this study, participants were recruited were recruited from three hospitals in Liaoning, Shanxi, and Beijing, China by means of convenience sampling. Prior to data collection, the team members responsible for the interviews received uniform training on how to use standardized language and instructions. The treatment and rehabilitation of older adult/adults patients after hip fracture is a long-term process, with postoperative functional recovery time ranging from 6 months to 1 year. Although the ideal time for functional recovery is 3 months after surgery, only 40 to 70 percent of patients regain their prior independence in activities of daily living within 6 months after fracture, and most patients still require long-term care (9, 23). older adult/adults patients with hip fracture have poor resistance to external stimulation, and the huge trauma of hip fracture and the operation itself makes the recovery of the self-care ability of older adult/adults patients with hip fracture face many problems (24). After discussion by the research team, patients who had undergone hip fracture surgery within 3 months to 18 months were selected as the study subjects. Finally, a total of 502 older adult/adults patients with post-operative hip fractures were recruited to participate in the study. The inclusion criteria required participants to be over 65 years of age; patients who had undergone hip fracture surgery within 3 months to 18 months; patients who were able to walk and have normal balance before hip surgery; clear consciousness and normal verbal communication; gave knowledgeable consent and willingly participated in the study. Patients who were bedridden and unable to communicate verbally due to stroke, dementia, or mental and cognitive disorders were excluded.

### 2.2. Instruments

#### 2.2.1. General information

The level of postoperative self-care for older adult/adults hip fracture patients is influenced by a number of factors. Studies have shown that differences in age, gender, functional status, fracture type, and other aspects of older adult/adults hip fracture patients can affect their level of post-operative self-care (25–27). The incidence of hip fractures increases with age and women are more likely to suffer a hip fracture than men. Different fracture types and functional statuses likewise influence the level of patient self-care after surgery. The researcher reviewed the literature and based on the content and purpose of this study and the clinical characteristics of the older adult/adults after hip fracture. A general demographic questionnaire was developed through an in-group discussion design. Participants were asked to self-report on nine factors, including age, sex, education, family type, occupation status, functional status, type of HFS, comorbidity, and time after surgery.

#### 2.2.2. The self-care scale for older adults undergoing hip fracture surgery

The scale was developed by Jeon et al. in 2022 to assess the level of self-care in older adults after hip fracture surgery (21). The scale included functional independence (4 items), symptom recognition and management (4 items), positive mental health (3 items), participation in and support for social activities (3 items), and safe environment (3 items). The scale consists of 5 dimensions and 18 items. Each item was rated on a Likert 5-point scale, from “never or very rarely” to “always” assigning a score of 1–5. The overall score varied from 18–90, with higher scores demonstrating higher levels of self-care among seniors undergoing hip fracture surgery. In the process of cross-cultural debugging, the item “Religion is very helpful for my recovery” was deleted, and the Chinese version of the HFS-SC with 5 dimensions and 17 items was finally formed.

### 2.3. Procedure

#### 2.3.1. Questionnaire translation procedure

Our translation work was approved by Professor Jeon. Firstly, the HFS-SC was translated by two bilingual Chinese native speakers. The parts that differed significantly were discussed by the research team and eventually agreed upon. The questionnaire was then counter-translated into English by two individual indigenous English speakers with Chinese language skills, and the translated scales were compared and modified by group discussion. In addition, translation experts were invited to culturally adapt the translated scale to make it more appropriate to Chinese language expressions. Finally, ten older adult/adults patients with hip fractures were recruited to take an initial survey using the translated scale. Participants were asked to complete the scale and give their opinions on the difficulty of the scale. Adjustments were made accordingly based on the participants' comments to ensure that the entries were semantically unambiguous, resulting in a final version of the Chinese version of the HFS-SC.

#### 2.3.2. Data collection procedure

After the training, the research team traveled to three cities and recruited participants using convenience sampling. Before the data survey, the research team explained the purpose of the survey to the subjects and ensured that the survey data was only used for academic research and would not be disclosed or used for other purposes without their permission. After the patients completed the questionnaire, the research team checked and collected the questionnaire. After eliminating invalid questionnaires, a total of 502 completed questionnaires were eventually collected. All data were collated, numbered and entered uniformly using the two-person entry method, and the entered data were reviewed to strictly ensure the accuracy of the data information. The principle of confidentiality was strictly followed during the study, and no identifiable information related to patients was involved in the data analysis. The privacy of the research subject was respected during the study and all information collected was kept confidential, and the researcher was not allowed to share private information about the subject without his or her consent. To assess retest reliability, 30 patients were asked to finish the post-translation scale again two weeks later.

##### 2.3.2.1. Data analysis

Data from this study were analyzed statistically by SPSS 26.0 and AMOS 26.0. Counting information was expressed as mean (standard deviation) and measurement information as percentage. P < 0.05 was considered statistically significant.

##### 2.3.2.2. Items analysis

Item analysis was designed to determine the discrimination and relevance of the scale, and the results could be used as a basis for item selection or modification. In item analysis, the scale was divided into the top 27% (high group) and the bottom 27% (low group) of the total score, and the relationship between the two groups was analyzed to determine whether the scale had sufficient discriminating ability. The critical ratio (CR) > 3.0 indicates good differentiation among items (28). The correlation coefficient between each item and the total score is analyzed to reflect the correlation between each item and the whole scale. *r* > 0.4 indicates good differentiation of each item. If each item is deleted, Cronbach's α coefficient through the aggregate table does not increase, and the item is retained. If Cronbach's α coefficient increases, it means that the measured attributes of this item are different from those of other items and should be removed.

##### 2.3.2.3. Reliability analysis

Reliability was an estimate of the consistency of the measurement. The higher the consistency of the measurement results, the higher the reliability of the tool (29). Reliability had characteristics such as internal consistency and stability. The indexes that often reflect the internal consistency of research tools were split-half reliability and Cronbach's α coefficient. The scale items were divided into two parts in parity order, and the split-half reliability was evaluated by calculating the correlation between the two parts. Retest reliability was often used to indicate the stability of a research tool (30). Two weeks later, 30 patients were selected for retesting as a way of assessing the stability and consistency of the scale across time.

##### 2.3.2.4. Validity analysis

Validity was to judge the accuracy of the content measured by the scale. This study tested the validity from two aspects: content validity and structure validity. Seven experts in related fields were invited to evaluate the content validity of the scale using the Delphi method. Each item was divided into four levels: no correlation (0 point), weak correlation (0 point), strong correlation (1 point) and strong correlation (1 point). The content validity index of the items (I-CVI) was the ratio of the number of experts who ranked a rating of 1 per project to the total number of experts. The content validity index of the scale (S-CVI) was the calculation result of the mean of I-CVI for each item of the scale. When I-CVI > 0.800 and S-CVI > 0.900, the content validity of the scale was better, and the items of the scale were more relevant and representative (31). Exploratory factor analysis (EFA) and confirmatory factor analysis (CFA) were used to assess the underlying factor structure of the questionnaire. A total of 502 samples were randomly divided into two groups with 251 participants in each group and were used independently for EFA and CFA. Data were considered suitable for principal component analysis only if the Bartlett's spherical test difference reached the significant level (*P* < 0.05) and the KMO was >0.6. The AMOS was used in CFA to analyze the suitability of model fitting indicators. Based on the maximum likelihood estimation, the model fitting index was determined by the following indicators: Chi-square degree of freedom (χ^2^/df) < 3.0, the goodness of fit index (GFI) > 0.9, the parsimonious goodness of fit index (PGFI) > 0.5, the incremental fit index (IFI) > 0.9, the root mean square error of approximation (RMSEA) < 0.08 indicates that model approximation is good and acceptable (32).

### 2.4. Ethical approval

Each participant was informed of the purpose and significance of the study and completed an informed consent form. Personal information was protected in the returned questionnaire. The study was approved by the Ethics Committee of Jinzhou Medical University and the process followed the ethical guidelines provided by the Ethics Committee.

## 3. Results

### 3.1. Descriptive statistics

A total of 502 older adult/adults patients were enrolled after hip fracture surgery: the mean age of participants was 72.88 years, and there were more females than males, 197 males (39.2%) and 305 females (60.8%). Most of the participants lived in couples, and 71.7% of the patients were retired. In terms of the type of surgery, 56.6% of the patients underwent total hip replacement (Table 1).

**Table 1:** General demography data (*n* = 502).

**Factors**	**Group**	***n* or M ±SD**	**%**
Age	65–74	344	68.5
	75–84	142	28.3
	≥85	16	3.2
Sex	Male	197	39.2
	Female	305	60.8
Education level	Primary school and below	261	52.0
	Junior high school	208	41.4
	Senior Secondary and above	33	6.6
Family type	Spouse	230	45.8
	Spouse+ offspring	88	17.5
	Solitary	75	14.9
	Offspring	109	21.7
Work	Part—time	35	7.0
	Full—time	360	71.7
	None	107	21.3
Functional status	Walking independently	348	69.3
	Walking with assistive device	154	30.7
Type of HFS	THR(Total Hip Replacement)	284	56.6
	BHA(Bipolar Hip Arthroplasty)	109	21.7
	PFNA(Proximal Femoral Nail Anti-rotation)	79	15.7
	(Open Reduction Internal Fixation)	30	6.0
Comorbidity	Present	154	30.7
	None	348	69.3
Period of HFS (months)	3–6	132	26.3
	7–12	192	38.4
	13–18	178	35.3
		12.3 ± 5.2	35.6

### 3.2. Intercultural adaptation

Due to the differences in language habits and cultural backgrounds of different countries, the scale needs to fully understand the Chinese context and thinking mode in the process of development. Therefore, the translation of the scale needs to be adjusted across cultures to make it more suitable for the target population. After obtaining the consent of the original authors, the research team made cross-cultural adjustments to the source scale. During the survey, the number of older adult/adults post-operative hip fracture patients who possessed religious beliefs was small and most held materialistic ideas. Many were mostly not devout believers and were reluctant to admit their beliefs in front of the collectors. During data collection, over 90% of the study participants selected “never or very rare” for this item. Considering the cultural differences in religious beliefs between the two countries, in the cultural background of China, the number of older adult/adults patients with religious beliefs after hip fracture surgery is small. Based on expert opinion and panel discussion, the item “Religion is very helpful for my recovery” was deleted after the group discussion. Then, nursing experts in geriatric nursing, rehabilitation nursing, psychometrics and other fields were invited to conduct expert correspondence consultation. Finally, a 5-dimensional, 17-item Chinese version of HFS-SC was formed.

### 3.3. Item analysis

The critical ratio (CR) of each item in the questionnaire ranged from 8.457 to 15.533, all of which were > 3. The score of each item was positively correlated with the total score (r = 0.446–0.629, *P* < 0.001). After deleting each item, the Cronbach's α coefficient of the scale was 0.835–0.845, which did not exceed the original Cronbach's α coefficient of 0.848 (Table 2).

**Table 2:** Item analysis for Chinese version of the self-care scale for older adults undergoing hip fracture surgery.

**Item**	**Critical ratio**	**Correlation coefficient between item and total score**	**Cronbach's Alpha if item delete**
1	11.780	0.542	0.839
2	12.438	0.581	0.838
3	13.608	0.572	0.838
4	14.382	0.592	0.837
5	8.457	0.446	0.845
6	15.487	0.629	0.835
7	14.973	0.609	0.836
8	12.402	0.514	0.841
9	9.715	0.493	0.843
10	10.918	0.526	0.841
11	12.492	0.544	0.839
12	13.434	0.515	0.841
13	13.463	0.534	0.840
14	9.866	0.464	0.845
15	14.988	0.575	0.838
16	13.024	0.532	0.840
17	15.533	0.554	0.840

### 3.4. Reliability analysis

The Cronbach's alpha coefficient for the translated scale was 0.848 and the Cronbach's alpha coefficient for each dimension ranged from 0.719 to 0.780. Two weeks later, 30 participants were randomly selected for retesting and the retest reliability for the translated scale was 0.759. The split-half reliability of the scale was 0.739.

### 3.5. Validity analysis

#### 3.5.1. Content validity analysis

Seven experts in related fields were invited to assess the content validity of the translated scale. The I-CVI of the Chinese version of the HFS-SC was 0.857 to 1 and the S-CVI was 0.932.

#### 3.5.2. Exploratory factor analysis

**Figure 1:**
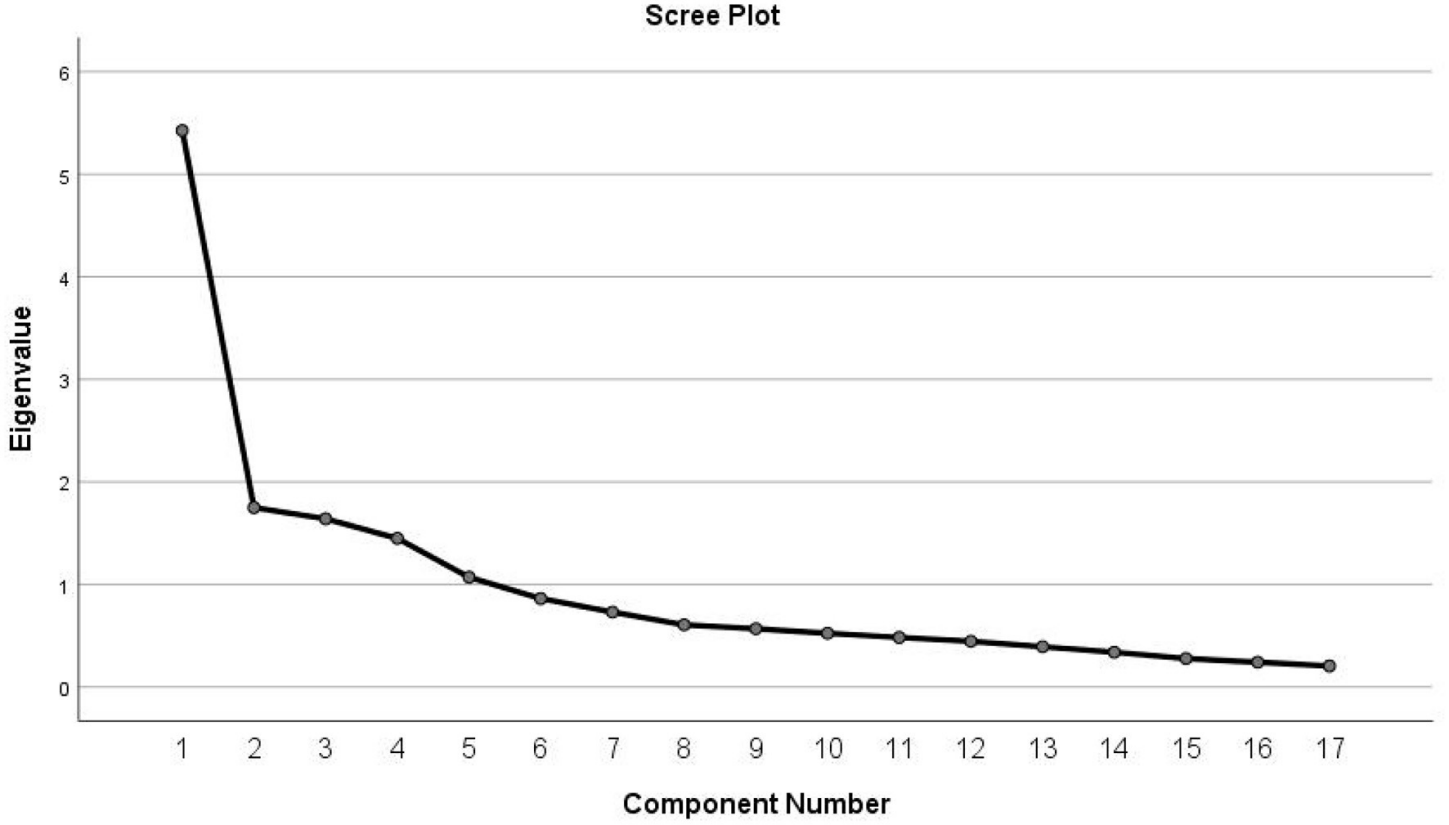
Screen plot of exploratory factor analysis for Chinese version of the self-care scale for older adults undergoing hip fracture surgery.

In this study, the KMO value was 0.804 and the Chi-square value of the Bartlett sphericity test was 1,668.585 (*P* < 0.001), indicating that the post-translated scale was suitable for factor analysis. Five common factors with eigenvalues > 1 were extracted based on the orthogonal rotation of principal component analysis and maximum variance method (Table 3), and 66.666% of the total variation was explained by the 5-factor model supported by the scree plot (Figure 1).

**Table 3:** Factor loadings of exploratory factor analysis for Chinese version of the self-care scale for older adults undergoing hip fracture surgery.

**Factor loading**	**Factor 1 (Functional independence)**	**Factor 2** **(Symptom recognition and management)**	**Factor 3** **(Positive mental health)**	**Factor 4** **(Participation in and support for social activities)**	**Factor 5** **(Safe environment)**
1	0.769	–	–	–	–
2	0.765	–	–	–	–
3	0.748	–	–	–	–
4	0.619	–	–	–	–
5	–	0.804	–	–	–
6	–	0.781	–	–	–
7	–	0.757	–	–	–
8	–	0.757	–	–	–
9	–	–	0.806	–	–
10	–	–	0.752	–	–
11	–	–	0.710	–	–
12	–	–	–	0.847	–
13	–	–	–	0.837	–
14	–	–	–	0.615	–
15	–	–	–	–	0.859
16	–	–	–	–	0.837
17	–	–	–	–	0.467

#### 3.5.3. Confirmatory factor analysis

**Figure 2:**
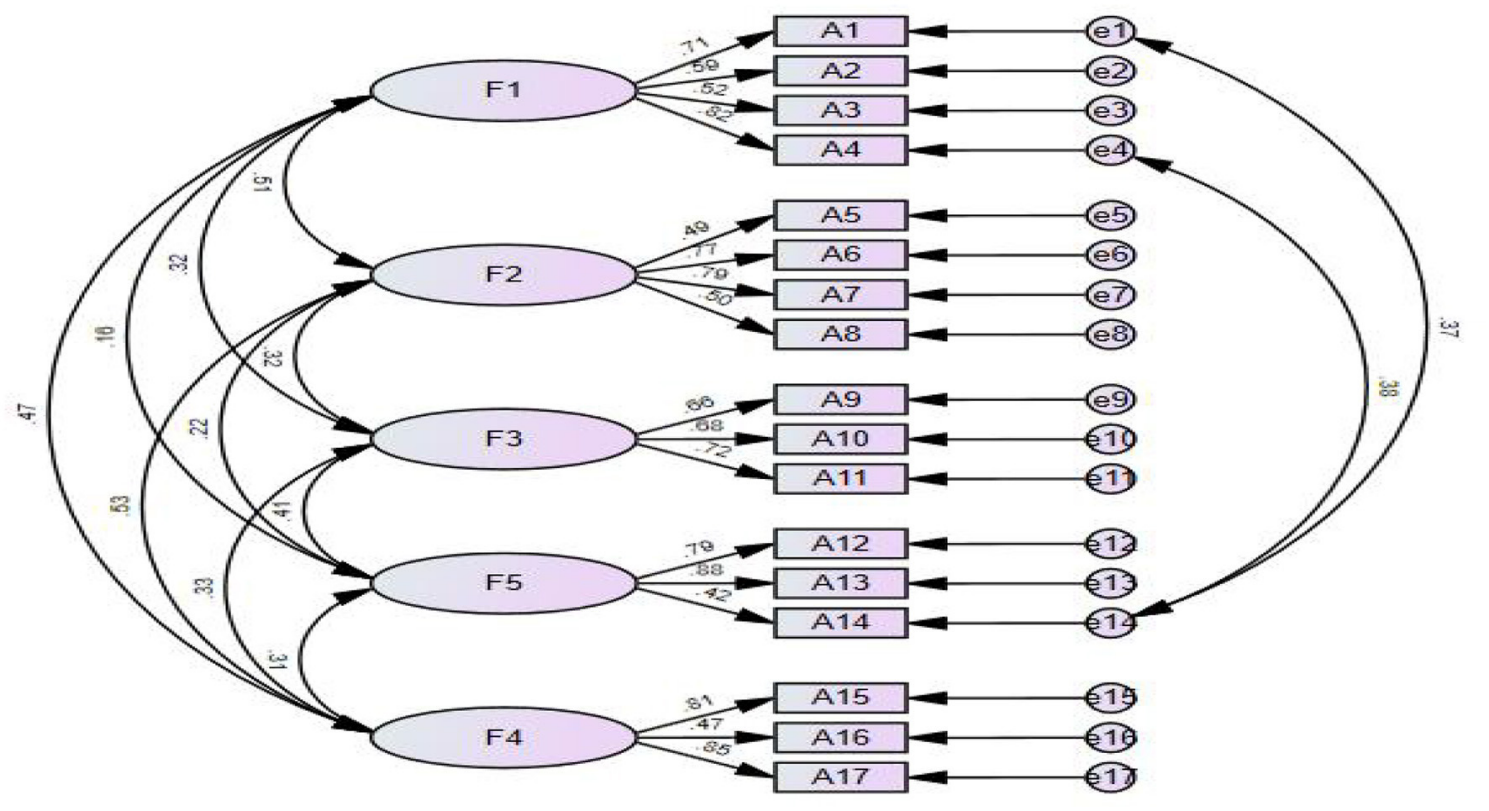
Standardized five-factor structural model of the self-care scale for older adults undergoing hip fracture surgery.

Based on the 5-factor structural model, the CFA was performed using AMOS software according to the maximum likelihood estimation (Figure 2). According to the Modification Index (MI), the initial model was modified by adding two residual paths: e1 and e14, and e4 and e14. After modification, the results of each fitting index showed that the X2/df = 1.847, GFI = 0.914, AGFI = 0.878, PGFI = 0.640, IFI = 0.932, TLI = 0.912, RMSEA = 0.058, PNFI = 0.679, CFI = 0.931. All fitting indexes of confirmatory factor analysis were within the reference range.

## 4. Discussions

### 4.1. The Chinese version of HFS-SC has suitable application value

The rate of aging is increasing globally as both the global economy and medical science grow. Fracture is now more prevalently recognized as a public health issue that reduces older adult/adults people's quality of life and healthy life span. One of the most dangerous fracture types is hip fracture. There is a lot of concern because of its abrupt start, protracted persistence, and high rates of disability and mortality (33). In China, the majority of old people reside far from their children, making recuperation more challenging and limiting their capacity to care for themselves. According to studies, patients' health can be considerably improved and quick recovery can be facilitated by having effective self-care abilities (20). Therefore, in order to achieve a healthy aging strategy, researchers should give the target group of older adult/adults hip fracture patients their undivided attention, promote self-care knowledge, assist patients and their families in bettering their disease awareness and daily behavior, and further improve the level of self-care ability of older adult/adults hip fracture patients.

### 4.2. The Chinese version of the questionnaire has suitable distinction

In accordance with the Brislin translation principles, experts in related fields were invited to debug the first draft of the translation (34). The preliminary and main survey showed that the translated scale is clearly expressed semantically and easy to understand. The Chinese version of the HFS-SC has 17 items and 5 dimensions. As older people grow older, their ability to discriminate declines. Fewer items, easier for the older adult/adults to understand and answer. The older adult/adults were all able to answer the translated scale accurately, and the actual completion was good. Item analysis indicated that there was a high degree of differentiation among the items of the Chinese version of HFS-SC, and the items were highly correlated with the scale. The Cronbach's alpha coefficient for each item after deletion did not surpass the original value. This indicates that all 17 items in the Chinese version of the HFS-SC can be retained with good discrimination (Table 4).

**Table 4:** The self-care scale for older adults undergoing hip fracture surgery.

**Factor**	**Item**
Functional independence	I try to do my daily living by myself without any help.
	I take painkiller on prescription after checking the pain intensity.
	I try to be well-nourished.
	I regularly work out on a daily basis.
Symptom recognition and management	Continuous management is required for hip fracture.
	I know when to visit an emergency room.
	I know what postures or exercises I have to avoid after surgery.
	I regularly visit the clinic to check my medical condition.
Positive mental health	I am careful for not falling again.
	I try to manage my depression from the limitation to move by myself.
	I can deal with stress.
Participation in and support for social activities	I have a good relationship with family members, friends and neighbors, and often meet with them.
	I have a person who I can ask for help in need.
	I currently participate in economic activity.
Safe environment	I remove any objects that might obstruct the pathway in order not to trip over.
	I wear shoes with rubber sole that are easy to put on.
	I leave some lights on but not too bright to disturb my sleep.

### 4.3. The Chinese version of the questionnaire has suitable reliability

Reliability test is used to reflect the reliability of the scale results (29). This study tested the scale from three aspects: internal consistency reliability, split-half reliability, and retest reliability. The results show that the Cronbach's α coefficient of the Chinese version of the HFS-SC is 0.848, the Cronbach's α coefficient for each dimension is 0.719–0.780, and the split-half reliability is 0.739. The reliability of the retest after 2 weeks was 0.759 (35, 36). The scale has good internal consistency and stability across time, and the scale reliability is good.

### 4.4. The Chinese version of the questionnaire has suitable validity

Validity refers to the degree to which the thing to be measured can be effectively measured by the measuring instrument. This study mainly analyzes the validity of the scale from content validity and structure validity. Content validity reflects whether each item conforms to the purpose and requirement of measurement, while structure validity reflects the degree of agreement between the theoretical assumption and the actual measurement. The I-CVI of the Chinese version of the HFS-SC was 0.857 to 1 and the S-CVI was 0.932, which were higher than the reference value of content validity, and the content validity was good (37, 38). In this study, EFA showed that the Chinese version of the 5-factor explained 66.666% of the total variance, the factor attribution of all items was consistent with that of the original scale (21, 36), and the factor loadings for each item were >0.4. Ultimately, the Chinese version of the HFS-SC model was within an acceptable range for all fitness indicators. In conclusion, the Chinese version of the HFS-SC has appropriate content validity and structural validity.

## 5. Limitations

There were some limitations in this study worthy of attention and discussion. Due to the self-reported nature of this study, bias was inevitable. Although the sample size met the study criteria, the sample was limited to selected provinces in China and a multi-center large sample study across China is needed. However, we did not explore the factors that influence self-care after hip fracture in older adults. Therefore, this is important for our next step.

## 6. Conclusions

After translation and cross-cultural adaptation, the self-care scale for older adults undergoing hip fracture surgery has been successfully introduced into China with good reliability and validity. The Chinese version of the HFS-SC can be used to assess the level of self-care of senior citizens in China following hip fractures and to create educational initiatives and research interventions, both of which are crucial for fostering healthy aging.

## Data availability statement

The raw data supporting the conclusions of this article will be made available by the authors, without undue reservation.

## Ethics statement

The studies involving human participants were reviewed and approved by Ethics Committee of Jinzhou Medical University. The patients/participants provided their written informed consent to participate in this study.

## Author contributions

CZ performed the data analysis and wrote the article. FL conceived the study. HZ directed the study and made the modifications. YZ, PC, and MZ collaborated to collect the data for this study. All authors contributed to the article and approved the submitted version.
